# Case Report: Left bundle branch pacing in an amyloid light-chain cardiac amyloidosis patient with atrioventricular block

**DOI:** 10.3389/fcvm.2023.1333484

**Published:** 2024-01-11

**Authors:** Jiaqi Yu, Fanyi Kong, Peng Gao, Taibo Chen, Yongtai Liu, Zhongwei Cheng, Hua Deng, Jinzhi Lai, Lihua Zhang, Jingbo Fan, Jiaqi Wang, Xiaohan Qin, Keyue Sun, Jian Li, Quan Fang, Deyan Yang, Kang’an Cheng

**Affiliations:** ^1^ Department of Internal Medicine, Peking Union Medical College Hospital, Chinese Academy of Medical Sciences & Peking Union Medical College, Beijing, China; ^2^ Department of Gastroenterology, Peking Union Medical College Hospital, Chinese Academy of Medical Sciences & Peking Union Medical College, Beijing, China; ^3^ Department of Cardiology, Peking Union Medical College Hospital, Chinese Academy of Medical Sciences & Peking Union Medical College, Beijing, China; ^4^ Department of Hematology, Peking Union Medical College Hospital, Chinese Academy of Medical Sciences & Peking Union Medical College, Beijing, China

**Keywords:** cardiac amyloidosis, conduction system disease, atrioventricular block, left bundle branch pacing, case report

## Abstract

**Introduction:**

Amyloid light-chain cardiac amyloidosis is a progressive infiltrative disease characterized by the deposition of amyloid fibrils in the cardiac tissue, which can cause serious atrioventricular block requiring pacemaker implantation. Left bundle branch pacing has emerged as an alternative method for delivering physiological pacing to achieve electrical synchrony of the left ventricle. However, left bundle branch pacing in patients with amyloid light-chain cardiac amyloidosis has not been studied in detail. Therefore, in this study, we present a case of left bundle branch pacing in a patient with amyloid light-chain cardiac amyloidosis.

**Case summary:**

A 66-year-old male patient with amyloid light-chain cardiac amyloidosis presented with syncope for 1 month. Holter monitoring revealed intermittent third-degree atrioventricular block. Left bundle branch pacing was performed successfully. During the 1-year follow-up, it was observed that the left bundle branch capture threshold remained stable without any pacemaker-related complications or left ventricle systolic dysfunction, and there was no recurrence of syncope.

**Conclusion:**

Left bundle branch pacing appears to be a safe and feasible option for patients with amyloid light-chain cardiac amyloidosis experiencing atrioventricular block.

## Introduction

Amyloid light-chain cardiac amyloidosis (AL-CA) is a progressive infiltrative disease characterized by the extracellular deposition of misfolded proteins in the cardiac tissue (1, 2). In addition to the most common manifestation of heart failure with preserved ejection fraction, various tachy- and bradyarrhythmias and conduction system diseases, such as atrial fibrillation, ventricular tachycardia, sinus node dysfunction (SND), atrioventricular block (AVB), and bundle branch block (3, 4), can be seen in AL-CA patients. A pacemaker should be implanted to correct serious SND and AVB (5). Pacemaker implantation is a common intervention among patients with cardiac amyloidosis (6). Conventional right ventricular apex pacing may result in left ventricular dysfunctions because of electrical dyssynchronization (7–9). Conduction system pacing, including left bundle branch pacing (LBBP), has been persuasively adopted in clinical practice to achieve electrical and mechanical synchrony of the left ventricle (10, 11). Nevertheless, there is limited evidence regarding the feasibility and safety of LBBP in AL-CA patients and whether the deposition of amyloid fibrils in cardiac tissue might cause pacemaker-related complications, such as an increase in the capture threshold leading to a loss of conduction system capture or ventricular undersensing, remains largely unknown. In the present study, we report a case of LBBP application in an AL-CA patient.

## Case presentation

**Figure 1:**
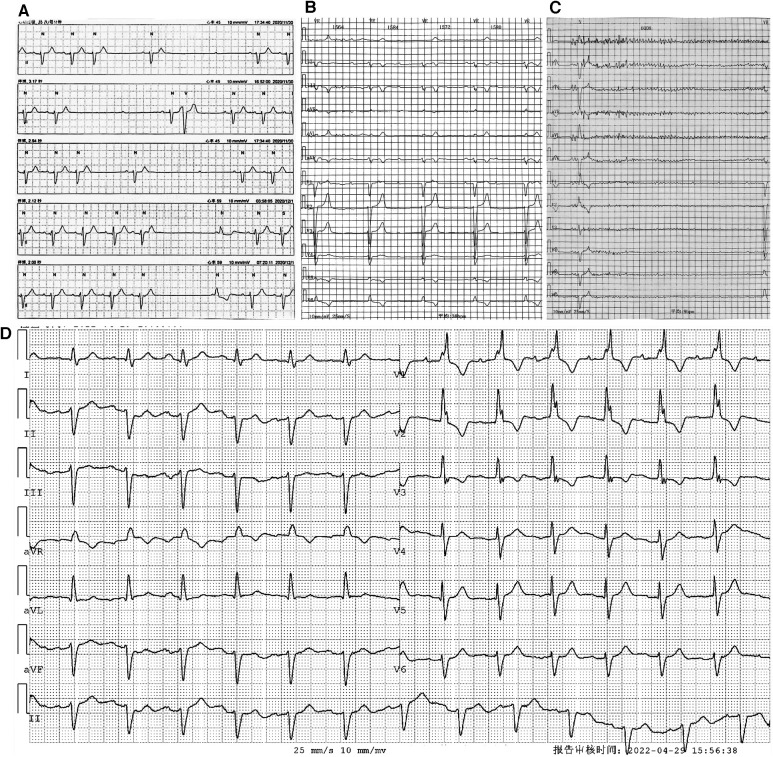
Holter monitoring of the patient. Holter monitoring in December 2020 (**A**) revealed an intermittent sinus pause and AVB. Holter monitoring after the first syncope episode in April 2022 (**B,C**) indicated an intermittent third-degree AVB with (**B**) and without (**C**) ventricular escapes. ECG without third-degree AVB (**D**) showed first-degree AVB, and the morphology of the QRS complex showed an RBBB pattern.

A 66-year-old man presented to our hospital on 13 May 2022 with recurrent syncope for 1 month. He was diagnosed with AL-CA 16 months previously, on 4 January 2021, when he presented with dyspnea on exertion. His blood pressure was recorded at 85/55 mmHg, and a physical examination revealed skin bruising and macroglossia. There was no evidence of polyneuropathy, dysautonomia, or carpal tunnel syndrome. Serum and urine protein electrophoresis with immunofixation revealed a positive result for monoclonal light-chain kappa, and the ratio of serum kappa:lambda free light chain (FLC ratio) was 79.46 (serum free light-chain kappa: 530.00 mg/L, and serum free light-chain lambda: 6.67 mg/L). The difference between involved and uninvolved free light chains (FLC-diff) was 52.33 mg/dl. The presence of amyloid deposits was confirmed by Congo red staining in the bone marrow biopsy tissue. Echocardiography revealed symmetry hypertrophy and diastolic dysfunction of the left ventricle (interventricular septum thickness: 12 mm, posterior wall of left ventricle thickness: 12 mm, left ventricular end-diastolic diameter: 42 mm, e′: 6.1 cm/s, and E/e′: 15.6) without pericardial effusion or aortic stenosis, and the left ventricular ejection fraction (LVEF) was 57%. Cardiac magnetic resonance (CMR) imaging indicated diffuse late gadolinium enhancement in the subendocardial regions and the involvement of the bilateral atrium, bilateral ventricle, and interventricular septum. Holter monitoring revealed an intermittent sinus pause and AVB, suggesting SND and conduction disease (Figure 1A). Per the result of the bone marrow biopsy, the presence of monoclonal light chains, and an abnormal FLC ratio, the diagnosis of light-chain amyloidosis was made. The confirmation of cardiac amyloidosis was based on the unexplained left ventricular thickness ≥12 mm and the diffuse late gadolinium enhancement in CMR (2). Based on the results of FLC-diff, cardiac troponin I (cTnI), and N-terminal pro-B-type natriuretic peptide (NT-proBNP) (FLC-diff: 52.33 mg/dl, cTnI: 0.04 μg/L, NT-proBNP: 1,021 pg/ml), the patient was classified as Mayo Stage Ⅱ (12), and BCD protocol chemotherapy, which included bortezomib, cyclophosphamide, and dexamethasone, was initiated on 8 January 2021.

After undergoing the ninth cycle of chemotherapy in September 2021, the patient achieved a very good partial response (VGPR) at the hematologic level, with a significant reduction in FLC-diff and FLC ratio from 52.33 to 1.55 mg/dl and from 79.46 to 2.24, respectively (13). In addition, cTnI and NT-proBNP decreased from 0.04 to <0.017 µg/L and from 1,021 to 541 pg/ml, respectively. Dyspnea was also relieved. Chemotherapy was suspended by his hematologist.

**Figure 2:**
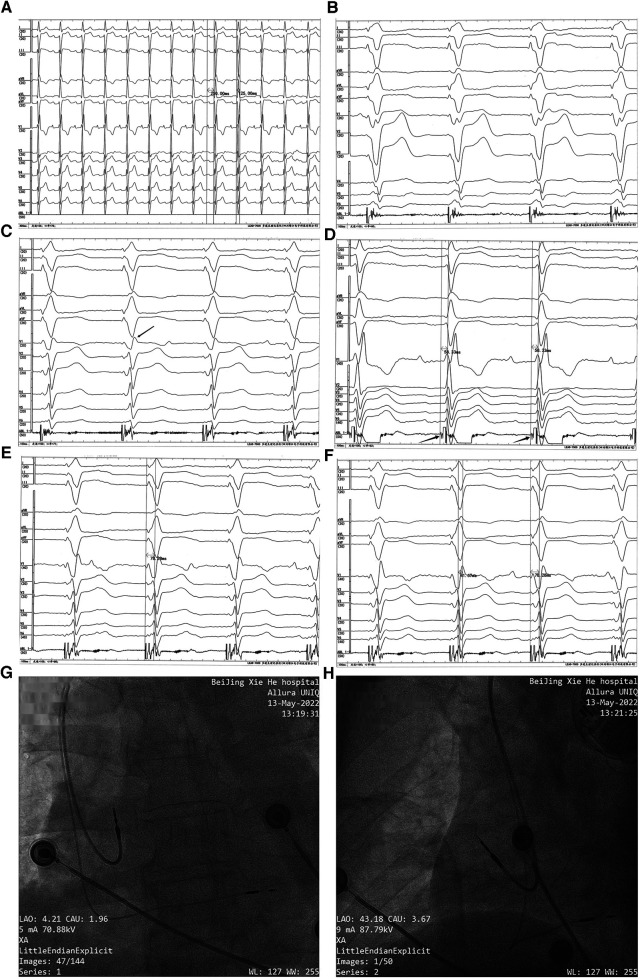
Permanent dual-chamber pacemaker implantation. Before the implantation procedure, the intrinsic ECG (**A**) showed first-degree AVB, RBBB, and LAFB (a sweep speed of 25 mm/s). Paced QRS morphology in the right ventricular septum (**B**) showed a “w” pattern with a notch at the nadir of the QRS in lead V1 (a sweep speed of 100 mm/s). With the lead screwed into the interventricular septum, the terminal R-wave of the paced QRS appeared in lead V1 (marked with a black arrow) (**C**) (a sweep speed of 100 mm/s). The left bundle branch potential (marked with a black arrow) was recorded from a Model 3830 lead tip (**D**) (unipolar, a sweep speed of 100 mm/s), and the left bundle branch potential to V6RWPT in the intrinsic rhythm was 58.33 ms. (**E**) Pacing at high voltage (5 V @ 0.4 ms) with the stimulus to V6RWPT of 78.28 ms (a sweep speed of 100 mm/s). (**F**) Pacing at low voltage (0.5 V @ 0.4 ms) with the stimulus to V6RWPT of 78.28 ms and a V6–V1 inter-peak interval of 41.67 ms (a sweep speed of 100 mm/s). (**G,H**) Positions of the pacing leads.

On 6 April 2022, the patient experienced his first syncope episode. Holter monitoring revealed an intermittent third-degree AVB with and without ventricular escapes (Figures 1B,C). During complete AVB, the heart rate was 38 bpm, and the morphology of ventricular escape exhibited a left bundle branch block (LBBB) pattern (QRS duration: 132 ms), indicating right bundle branch origination (Figure 1B). However, during atrioventricular 1:1 conduction, the PR interval was 220 ms, and the morphology of the QRS complex exhibited a right bundle branch block (RBBB) pattern, indicating conduction through the left bundle branch (Figure 1D). The results of repeated laboratory tests of FLC-diff showed stability without amyloidosis relapse, and the echocardiography results were similar to those of the previous one. On 13 May 2022, the patient was admitted to our hospital for further therapy. Because of the new-onset syncope and the worsening of atrioventricular conduction without reversible etiologies, a permanent dual-chamber pacemaker was planned to be implanted, and to achieve electrical and mechanical synchrony, an LBBP was attempted. Before the implantation procedure, the patient’s intrinsic electrocardiogram (ECG) showed a sinus rhythm with 1:1 atrioventricular conduction, and the QRS complex exhibited an RBBB pattern with a duration of 125 ms and without decreased voltage. Because the PR interval was 250 ms and the frontal plane axis showed a left deviation of −43°, consistent with a left anterior fascicular block (LAFB), a diagnosis of first-degree AVB and LAFB was confirmed, and supraventricular excitation was presumed to be conducted through the left posterior fascicle (Figure 2A).

The LBBP procedure was performed as previously described (14). The pacing lead (Model 3830; SelectSecure, Medtronic Inc., Minneapolis, MN, USA) was supported by the delivery sheath (C315His; Medtronic Inc.) via the left axillary vein. The paced ECG and the intracardiac electrogram were collected using an ECG recording system (LEAD-7000 C EP Recording System, Jinjiang Electronic Medical Device Technology Co., Ltd., Sichuan, China). The delivery sheath was advanced across the tricuspid valve summit in the right anterior oblique (RAO) view at 30° and was further advanced to approximately 20 mm toward the right ventricular apex. A counterclockwise torque was applied so that the sheath could reach the right ventricular base to the mid-septum. The initial pacing site was the right ventricular septum. The QRS morphology in the pacing site showed a “w” pattern with a notch at the nadir of the QRS in lead V1 (Figure 2B). Then, the lead was screwed into the interventricular septum until a paced QRS (voltage output at 5 V @ 0.4 ms in the unipolar mode) of RBBB morphology with a terminal R-wave in lead V1 was obtained (Figure 2C). There was little difficulty in screwing the Model 3830 lead into the deep septum. The left bundle branch potential could be recorded when the lead was deeply screwed into the subendocardial area on the left side of the interventricular septum (Figure 2D). A threshold test was performed, and it showed that the transitions in the QRS morphology were quite subtle when pacing from high voltage (5 V @ 0.4 ms) to low voltage (0.5 V @ 0.4 ms); therefore, the test was non-conclusive. When pacing at high and low output voltages, consistent values of the stimulus to V6 R-wave peak time (V6RWPT) were recorded, as shown in Figures 2E,F. To confirm the left bundle branch capture, the LBBP ECG criteria were evaluated. The V6–V1 inter-peak interval was 41.67 ms (Figure 2F), and the stimulus to V6RWPT was 78.28 ms (Figure 2F). Because the left bundle branch potential to V6RWPT in intrinsic rhythm was 58.33 ms (Figure 2D), the difference between the potential to V6RWPT and the stimulus to V6RWPT was 19.95 ms. Because V6RWPT <80 ms fulfilled the European Heart Rhythm Association (EHRA) criteria (14), the LBBP of our patient was confirmed. The threshold of left bundle branch capture measured in the unipolar mode was 0.5 V @ 0.4 ms, the R-wave amplitude measured in the bipolar mode was 21.0 mV, and the ventricular lead impedance was 774 Ω. The threshold of right atrium capture was 0.8 V @ 0.4 ms, the P-wave amplitude was 1.6 mV, and the atrial lead impedance was 922 Ω. The paced QRS duration was 106 ms. The pacing leads were connected to a generator (X3DR01 Astra S DR, Medtronic Inc.). The positions of the leads are shown in Figures 2G,H.

**Figure 3:**
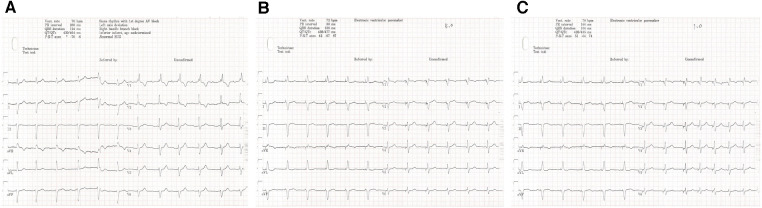
One-year follow-up visit. The intrinsic rhythm (**A**) was a sinus rhythm with first-degree AVB. The stimulus to V6RWPT (78 ms) was stable at high (5.0 V @ 0.4 ms) (**B**) and low (1.0 V @ 0.4 ms) (**C**) output voltages when the left bundle branch was captured.

After pacemaker implantation, there was no occurrence of recurrent syncope. At the patient’s 3-month follow-up visit, the pacing parameters were stable. The unipolar left bundle branch capture threshold was recorded at 0.75 V @ 0.4 ms, the unipolar pacing impedance was measured at 570 Ω, and the sensed bipolar R-wave amplitude was 20.0 mV. The right atrium capture threshold was recorded at 0.75 V @ 0.4 ms, the pacing impedance was measured at 323 Ω, and the sensed P-wave amplitude was 1.1 mV. At the 1-year follow-up visit, the patient's intrinsic rhythm was a sinus rhythm with first-degree AVB (Figure 3A); therefore, the left bundle branch capture threshold was measured by mandatory ventricular pacing, and the value was 0.75 V @ 0.4 ms. The sensed R-wave amplitude and pacing impedance were 20.0 mV and 532 Ω, respectively. The right atrium capture threshold was recorded at 1.0 V @ 0.4 ms, the pacing impedance was measured at 323 Ω, and the sensed P-wave amplitude was 0.8 mV. The stimulus to V6RWPT (78 ms) was stable at high (5.0 V @ 0.4 ms) and low (1.0 V @ 0.4 ms) output voltages when the left bundle branch was captured (Figures 3B,C). The atrial and ventricular pacing rates were 37.4% and 7.7%, respectively. In addition, no pacemaker-related complications, such as pocket infection, hematoma, lead dislodgement, and rupture, were reported. There was no reported occurrence of syncope, and the patient did not experience dyspnea on exertion.

**Figure 4:**
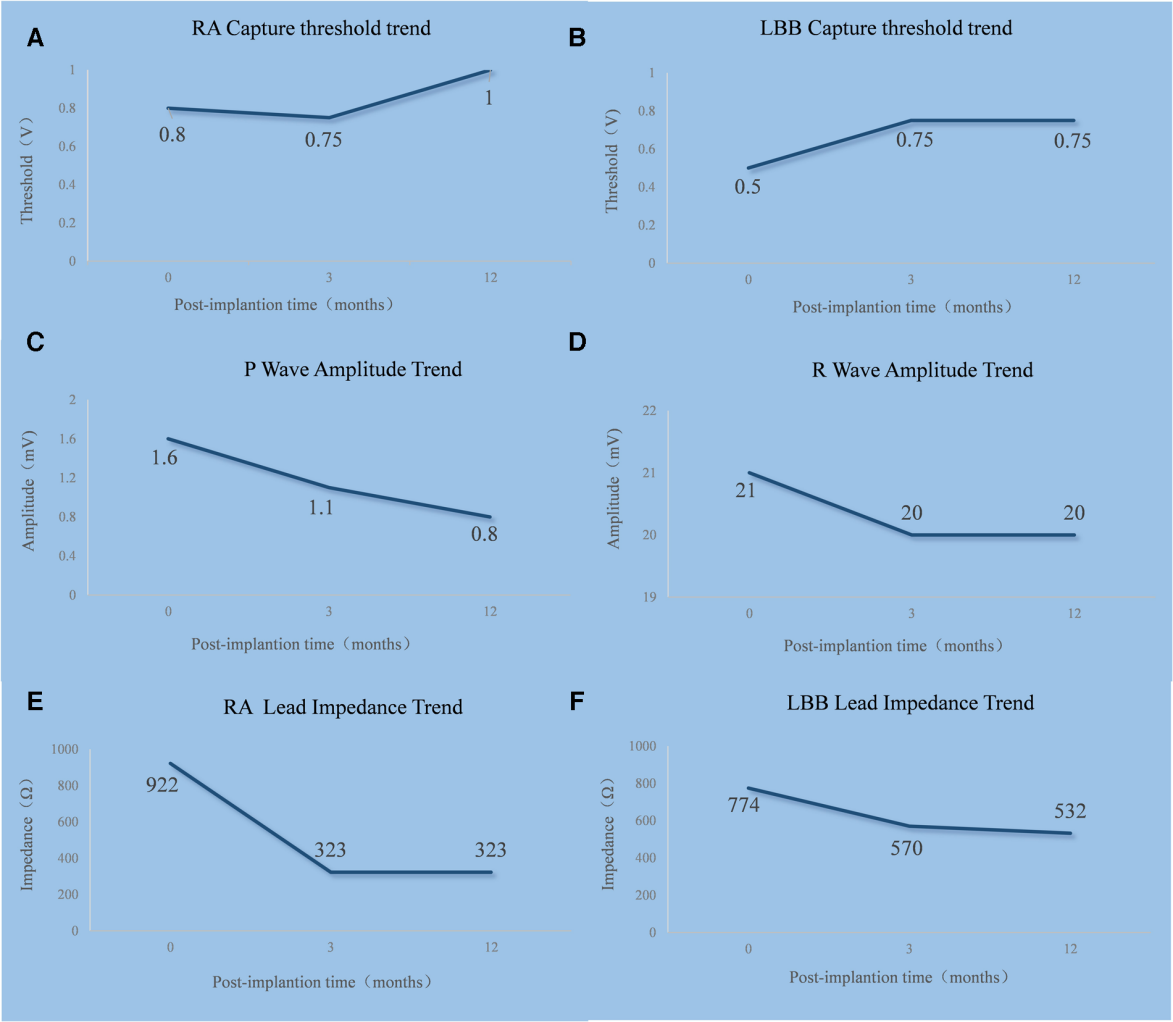
Trends in the RA lead (**A,C,E**) and LBBP lead (**B,D,F**) threshold, sensing, and impedance. RA, right atrium; LBB, left bundle branch.

The trends in the right atrial lead and LBBP lead threshold, sensing, and impedance are shown in Figure 4. The timeline of the present case is shown in Supplementary Table 1.

## Discussion

Conduction system diseases are not uncommon in AL-CA patients. In a study of 157 AL-CA patients, first-degree AVB, RBBB, LBBB, and LAFB were present in 18%, 19%, 4%, and 29% of the patients, respectively (15). Recently, a large cohort study showed that 8.9% of the patients with cardiac amyloidosis received a pacemaker because of SND or AVB within 3 years after diagnosis (6). The pathogenesis of conduction system diseases in cardiac amyloidosis is multifactorial, including amyloid deposition causing a disruption of the transmission of electrical impulses along the conduction fibers and the cytotoxicity of amyloid precursor proteins (3).

In the general population with right ventricular pacing, a significant decline in LVEF was noted, and the incidence of pacing-induced cardiomyopathy was also significantly higher (16). Among patients with amyloid transthyretin cardiac amyloidosis (ATTR-CA) who have implantable devices, a higher conventional right ventricular pacing burden is associated with deleterious remodeling and congestive heart failure (9). Conduction system pacing techniques, such as His bundle pacing and LBBP, have been demonstrated to be superior to conventional right ventricular apex pacing because they achieve electrical synchrony of the left ventricle (7, 8). However, evidence of LBBP in infiltrative cardiomyopathy mainly caused by amyloidosis is scarce.

In the present case, LBBP was successfully used in an AL-CA patient with third-degree AVB. During the patient’s 3-month and 1-year follow-up visits, no occurrence of pacemaker-related complication was reported, and the left bundle branch capture threshold, R-wave amplitude, and ventricular lead impedance remained stable. Although the extracellular deposition of amyloid fibrils in the myocardium and conduction system might potentially cause an increase in the ventricular pacing capture threshold, and the frequently encountered decreased QRS voltage might cause device undersensing, a previous retrospective observational study of 34 patients with AL- or ATTR-CA and a cardiac implantable electronic device showed that the mean right ventricular capture threshold (0.8 ± 0.1 V at 3 years) and the mean right ventricular lead impedance (418 ± 28 Ω at 3 years) remained stable over a mean follow-up of 3.1 ± 4.0 years, but the mean right ventricular sensing was 10.6 ± 0.8 mV at 1 year, which decreased to 6.7 ± 1.2 mV at 3 years without resulting in device malfunction (17). Taha et al. reported a case of an ATTR-CA patient with LBBP who demonstrated a significant alleviation of heart failure symptoms and improvement in exercise capacity during 3- and 6-month follow-up visits with stable pacing thresholds (18). The lead parameters of our patient at follow-up were consistent with those of the previous studies (17, 18). Recently, Chinh et al. reported a retrospective cohort including 23 patients with ATTR-CA or AL-CA who underwent left bundle branch area pacing for bradycardia or cardiac resynchronization therapy. The sensed R-wave amplitude and pacing threshold were stable during a mean follow-up time of 7.7 months (19). Using stricter criteria of LBBP in our patient, we also found stable pacing parameters during follow-up visits, which was consistent with a study by Chinh et al. We speculated that the conduction capacity of the left bundle branch might be partially preserved in some AL-CA patients and that the left bundle branch could be captured with an acceptable threshold. LBBP appears to be a promising pacing modality in AL-CA patients requiring a pacemaker. However, further studies are needed to determine the long-term feasibility and safety of applying LBBP in AL-CA patients and to determine its effect on left ventricular function.

## Conclusion

AL-CA with serious AVB might be a potential candidate for the application of LBBP. Further studies are needed to determine the long-term feasibility and safety of the application of LBBP in patients with AL-CA and to ascertain its effect on left ventricular function.

## Limitations

There are several limitations in our study. First, an invasive electrophysiological study was not performed and we could not confirm the level of conduction block. Second, the baseline LVEF of the patient was normal and the ventricular pacing percentage was quite low; therefore, the present findings cannot be generalized to patients with left ventricular systolic dysfunction and high-burden ventricular pacing. Finally, syncope and a worsening of AVB occurred after light-chain amyloidosis recovery; therefore, other factors contributing to the development of AVB could not be completely excluded. However, only 25%–50% of all patients treated with chemotherapy achieve an organ response (20). The present case indicated that atrioventricular conduction worsening could develop even in patients with hematological remission after chemotherapy.

## Data Availability

The original contributions presented in the study are included in the article/**Supplementary Material**, and further inquiries can be directed to the corresponding authors.
