# Supplementary material for: Case Report: Left bundle branch pacing in an amyloid light-chain cardiac amyloidosis patient with atrioventricular block
Source: Front Cardiovasc Med. 2024 Jan 11;10:1333484. doi: 10.3389/fcvm.2023.1333484 (PMC10808645; doi:10.3389/fcvm.2023.1333484)
Supplement: Supplementary file 1 [file Table1.docx]

Supplementary Material

# Supplementary Figures and Tables

Supplementary Table 1. Timeline of the present case

| December 2020 | Holter monitoring revealed intermittent sinus pause and AVB. |
| --- | --- |
| January 4th 2021 | AL-CA was diagnosed. |
| January 8th 2021 | BCD protocol chemotherapy started including bortezomib, cyclophosphamide and dexamethasone. |
| September 2021 | VGPR at the hematologic level after the 9th cycle of chemotherapy |
| April 6th 2022 | First syncope episode. A subsequent Holter monitoring revealed intermittent third-degree AVB with and without ventricular escapes. |
| May 13th 2022 | Permanent dual chamber pacemaker implantation with LBBP |
| August 2022 | 3-month follow-up visit with stable pacing parameters and no recurrent syncope |
| June 2023 | 1-year follow-up visit with left bundle branch capture and stable pacing parameters. No pacemaker-related complications were reported and no syncope or symptoms related to heart failure occurred. |

AL-CA, amyloid light chain cardiac amyloidosis; VGPR, very good partial response; AVB, atrioventricular block; LBBP, left bundle branch pacing.
